# A Treatment-Response Comparison Study of Resting-State Functional Magnetic Resonance Imaging Between Standard Treatment of SSRI and Standard Treatment of SSRI Plus Non-dominant Hand-Writing Task in Patients With Major Depressive Disorder

**DOI:** 10.3389/fpsyt.2021.698954

**Published:** 2021-09-03

**Authors:** Rabia Kevser Boyraz, Ismet Kirpinar, Onur Yilmaz, Onur Özyurt, Tezer Kiliçarslan, Ayse Aralasmak

**Affiliations:** ^1^Bezmialem VAKIF University, Department of Psychiatry, School of Medicine, Istanbul, Turkey; ^2^Bogaziçi University, Bogaziçi Engineering Institute, Istanbul, Turkey; ^3^Bezmialem Vakıf University, Department of Radiology, School of Medicine, Istanbul, Turkey

**Keywords:** major depressive disorder, resting-state functional magnetic resonance imaging, non-dominant hand-writing exercise, treatment response, comparison study

## Abstract

**Background:** Researches have recently shifted from functional/structural imaging studies to functional connectivity (FC) studies in major depressive disorder (MDD). We aimed to compare treatment response of two treatment groups before and after treatment, in terms of both with psychiatric evaluation scales and resting-state functional connectivity (RSFC) changes in order to objectively demonstrate the possible contribution of the non-dominant hand-writing exercise (NHE) effect on depression treatment.

**Methods:** A total of 26 patients who were right-handed women with similar sociodemographic characteristics were enrolled. Their pre-treatment resting-state functional magnetic resonance imaging (rs-fMRI) and neuropsychiatric tests were recorded, and then, patients were divided into two groups randomly. A standard treatment (ST) (fix sertraline 50 mg/day) was given to both groups. One randomly selected group was given the NHE in addition to the ST. After 8 weeks of treatment, all patients were reevaluated with rs-fMRI and neuropsychiatric tests. Pre- and post-treatment FC changes within the groups and post-treatment connectivity changes between groups were evaluated.

**Results:** Post-treatment neuropsychiatric tests were significantly different in both groups. Post-treatment, two brain regions' connectivity changed in the ST group, whereas 10 brain regions' connectivity changed significantly in the ST + NHE group. When treatment groups were compared with each other after the treatment, the FC of 13 regions changed in the ST + NHE group compared to the ST group (*p*-unc/*p*-PFD <0.05). The density of connectivity changes in the frontal and limbic regions, especially connectivities shown to change in depression treatment, in the ST + NHE group indicates a positive contribution to depression treatment, which is also supported by neuropsychiatric scale changes.

**Conclusion:** NHE, which we developed with inspiration from the Eye Movement Desensitization and Reprocessing (EMDR) method, showed significantly more connecitivity changes related with MDD treatment. Beyond offering a new additional treatment method, our study will also contribute to the current literature with our efforts to evaluate all brain regions and networks that may be related to MDD and its treatment together, without being limited to a few regions.

**Trial Registration:** The rs-fMRI and treatment registers were recorded in the BizMed system, which is the patient registration system of Bezmialem Vakif University Medicine Faculty, under the BAP support project approval code and the registration number 3.2018/8.

## Introduction

With increasing neuroimaging studies in major depressive disorder (MDD), it is seen that resting-state functional connectivity (RSFC) changes are among the reasons mentioned more in etiology rather than a localized brain region (1). In connectivity reviews focusing on MDD, default mode network (DMN), salience network (SN), and affective network (AN) have been shown to have impaired internal activity (2). DMN changes, which are thought to play a central role in MDD, have been reported to occur not only in patients but also in individuals at high risk of developing depression (3). Many studies are showing decreased connectivity in the frontal regions in MDD patients (4). Anand et al. have also shown increased connectivity between the anterior cingulate cortex (ACC) and limbic regions (thalamus, pallido-striatum, and amygdala) after 6 weeks of sertraline treatment (5). Similarly, an open-label sertraline treatment-response study found increased connectivity between the frontal and limbic regions and it contributed to a more effective inhibitory response in emotion regulation (6). In another RSFC study, it was shown that the connectivity from the posterior cingulate cortex (PCC) to the right lateral parietal cortex and right inferior temporal gyrus (ITG) returned to normal after duloxetine treatment (7). It has been shown that ACC is a predictor in MDD treatment response and ACC is a bridge between the dorsolateral prefrontal cortex (DLPFC) and sub-regions as an explanation. A high ACC regional homogeneity (ReHo) value can predict good top-down control.

### Potential Role of Eye Movement Desensitization and Reprocessing in MDD Treatment and Its Mechanism

Bergmann et al. showed that a successful eye movement desensitization and reprocessing (EMDR) provides a normalized functional balance between limbic and prefrontal areas and thalamic repair with increased thalamic activation (8). After EMDR, connectivity changes between the rostral region of ACC and the lower caudate region, which is more related to cognitive functions, are noteworthy. EMDR increases the activity of the hippocampus and amygdala by limiting the limbic system hyperactivity with the low-frequency eye stimulus, as well as bringing ACC activity to a normal level (9). According to Shapiro's adaptive information processing model (AIP), the brain narrates memories to make sense and save material presented by reality. The story creation module consists of DMN. Stressful events (even non-A criterion types) may be dysfunctionally stored, and these stressful memories may consequently form the basis of mental disorders like depression. High relapse rates of MDD are not only associated with specific personality traits and dysfunctional beliefs/schema but also with a history of trauma or critical life events.

A randomized clinical trial comparing the effectiveness of fluoxetine with EMDR treatment and placebo in a traumatized population found that the EMDR group have significantly lower depression scores than the fluoxetine group (10). A larger study investigated whether different results may be obtained in depressive patients without an explicit trauma history when adding additional EMDR therapy in comparison with cognitive behavioral treatment (CBT) treatment. The CBT + EMDR group showed more complete remissions and a greater reduction in Beck Depression Inventory scores than the CBT-only group (11). The retrieved researches suggest EMDR as a useful supplementary therapy not only in the treatment of MDD with and without co-concurrent trauma history but also in the case of recurrent, long-term symptomatology and treatment-resistant (TR) depression (10, 12). Overall, the studies that applied EMDR in MDD showed a reasonable level of evidence ranging from 2 to 5 on the Sackett Scale.

### Hemispheric Lateralization and Non-dominant Hand-Writing Exercise

Both hemispheres are asymmetric structurally and functionally from birth. Hand preference is used to understand lateralization roughly. Our right hand is directed by the left brain and our left hand by the right brain (13). Due to the hemispheric asymmetry, we include patients with similar hemispheric dominance (right-handed) to ensure the uniformity of resting-state functional magnetic resonance imaging (rs-fMRI) in this study. Besides, when the relationship between hemispheric lateralization and depression is examined, depressive symptoms are compatible with the right hemisphere dysfunction hypothesis, while the manic episode is compatible with right hemisphere hyperfunction (14). Connectivity abnormalities mainly occur in the right hemisphere, which is consistent with the previous studies showing the dominant role of the right hemisphere in depression. Related studies suggest that the physiological activation of the right hemisphere of the brain in depressive patients is accompanied by functional decline and deficiencies. The additional physiological activity in the right hemisphere is to overcome functional barriers. To be able to properly complete corresponding functions, the right brain of depressive patients must increase its activity level (15). Although hemispheric lateralization has not been proven in the etiology of depression, it is one of the mechanisms explaining the contribution and effectiveness of EMDR treatment in depression.

Although eye movement is especially emphasized in EMDR, it can also be applied in some patient groups by touching the right-left body area (arm and leg), respectively, when the eyes are closed. So, the aim is to stimulate both sides of the body. With the knowledge that the right-handed person constantly stimulates left brain motor areas in daily work, we think that the daily non-dominant hand-writing exercise (NHE) (with left hand) will increase the right hemispheric activity and contribute to the more balanced work of the two brain regions. Here, our aim is to develop an additional treatment method such as EMDR that can be applied to oneself at home for patients who do not have the opportunity to receive EMDR therapy.

### The Aim of the Study

In this study, we aimed to compare the treatment response of two treatment groups before and after treatment, in terms of both psychiatric evaluation scales and RSFC changes.

Our hypothesis is NHE produces an additional benefit over depression treatment as usual *via* achieving the integrity of the two hemispheres, increasing the control of the upper regions to the lower brain, and increasing right brain activity, like the EMDR effect. Our goal is to objectively demonstrate the possible contribution of the NHE effect on depression treatment with psychiatric scales and neuroimaging.

## Materials and Methods

### Neuropsychiatric Evaluation Scales

The neuropsychiatric evaluation scales used in this study are as follows:

**Beck Depression Inventory (BECK-D):** It consists of 21 self-assessment items developed by Beck et al. (16).
**Hamilton Depression Rating Scale (HAM-D):** The scale prepared by Hamilton in 1960 has 17 items. The Turkish version is developed by Akdemir et al. (17).
**Hand Preference Questionnaire:** It was developed by Chapman and Chapman in 1987 (18). It consists of 13 items that question which hand is preferred during various manual tasks. According to the total, individuals scoring 13–17 are defined as “right-handed,” individuals scoring 18–32 are “two-handed,” and individuals scoring 33–39 are “left-handed.”
**Frontal Assessment Battery (FAB):** It was developed by Dubois et al. to evaluate frontal functions (19). The total score ranges from 0 to 16 points, with cut-off values of 12 and above suggesting normal frontal functions.
**NHE**: Since the study sample consisted of right-handed individuals, it was deemed appropriate to give a left-hand writing task. So, we described the NHE as text consisting of two paragraphs of 50 words per day to be written with the left hand. To unify word selection for all ethnic groups, a volunteer research assistant from Marmara University Turkish Language and Literature Department prepared 120 paragraphs (38 of them are Turkish words, 37 of them are from Persian and Arabic, and 37 of them are from the European language family) to apply daily for 8 weeks.
**20-Item Toronto Alexithymia Scale (TAS-20)** It was developed by Bagby et al. to determine the severity of alexithymic symptoms. It was found appropriate to take 51 points as the cut-off value and 59 points as the upper value.

### Study Sample and Design

Having neurological or additional psychiatric disorder, having been receiving any psychiatric treatment for the past 2 months, being left-handed, being hearing and visually impaired, using a psychoactive substance, and having any obstacle to MRI are the exclusion criteria.

Volunteering was based on participation in this study. Regarding the treatment process of the participants, any changes were made during the study. Medical researchers were carried out within the framework of the Helsinki Declaration and Code of Ethics (T.C. Bezmialem Vakıf University Clinical Research Ethics Committee approved all steps of the study, no. 3.2018/8.)

Twenty-six volunteer female patients who were aged 18–50 years, right-handed, diagnosed as *unipolar* MDD according to SCID II-DSM 5, and accepted the informed consent by exceeding the exclusion criteria, same with the patient's group, were included in the study [upon the radiology specialist's recommendation, we chose patients of a single gender (female), with similar educational level (university graduate), and who were right-handed to provide rs-fMRI uniformity].

Neuropsychiatric tests and rs-fMRI were administered to all patients before starting treatment. Twenty-six patients were randomly divided into two groups, and all started a fixed 50-mg/day dose of sertraline to ensure medical treatment standardization in both groups. While the first group continued 50 mg/day sertraline only and was named as ST, the second group continued NHE daily in addition to 50 mg/day sertraline treatment and was named as ST + NHE group. Regarding the treatment process of the participants, any changes were made during the study for 8 weeks. At the end of the study, all patients were reevaluated with rs-fMRI imaging and neuropsychiatric tests. We compared both treatment groups within for pre- and post-treatment status and with each other for post-treatment status in terms of treatment responses.

### Statistical Analysis

Scale scores and sociodemographic data were analyzed with the IBM SPSS Statistics 20.0 (IBM, Armonk, NY, USA) package program. Considering the similar studies conducted in the literature about the variable values that will be used for the relevant study in the Department of Biostatistics of our university, a minimum sample of 22 people was determined when 95% confidence level, 80% power, and *p* < 0.05 significance were added. Based on this, we decided to have 26 people. The Wilcoxon signed-rank test was used for psychiatric evaluation score comparison within the groups and between groups.

### The Acquisition and Analysis of rs-fMRI Data

MRI data from the whole-brain parenchyma was taken by the radiology specialist physician in an average of 9 min with a 1.5-Tesla Siemens MRI device (Siemens, Munich, Germany) in the radiology department. Imaging results were obtained for each patient consisting of 205 volumes and 40 volumes per volume. Anatomical imaging is shown by the T1-weighted MPRAGE sequence. To perform image analysis with functional images, preliminary image processing procedures consisting of various phases were applied over the CONN software program using the MATLAB-based statistical parametric mapping program. Bandpass filter, first-level analysis, and second-level analysis were applied to the obtained images, respectively. Self-monitoring over functional images converted from 2D DICOM format to 3D.nii format was done with the following steps: realignment, co registration, normalization, segmentation, smoothing, and filtering.

With the help of a computer engineer from Bogaziçi University, MATLAB-based CONN functional connectivity toolbox was used for statistical analysis of rs-fMRI data. Region of interest (ROI)-to-ROI method was used for analysis. In the ROI–ROI maps, the functional connectivity map depicts the correlation between each ROI with every other ROI in the brain. The ROI–ROI results allow us to see in more detail how nodes of certain networks are correlated with other nodes in the brain.

Study groups were compared with each other in terms of the connectivity of 165 × 165 areas with the ROI–ROI method by taking 165 anatomic brain regions registered in the CONN system (https://web.conn-toolbox.org/conn-in-pictures).

Functional connectivities were compared to see the difference between the pre- and post-treatment conditions and between the two treatment groups, with *p* < 0.05 [both *p-*uncorrected (*p*-unc) and false discovery rate-corrected *p*-values (*p*-FDR) values were taken into consideration]. ROI–ROI connectivity changes of treatment groups were compared by two-sample tests.

## Results

### Comparison of Treatment Groups According to the Sociodemographic Data and Psychiatric Rating Scales

Treatment groups were statistically similar in terms of age, gender, and educational status.

After 8 weeks of treatment, the ST group's mean HAM-D score decreased significantly from 24.17 to 7.30 (*p*-value: 0.008), the mean BECK-D scores decreased significantly from 27.58 to 12.70 (*p*-value: 0.008), the mean FAB scores increased from 15.92 to 16.90 significantly (*p*-value: 0.034), and the total mean Toronto Alexithymia Scale (TAS) score decreased from 59.08 to 57.10, but the difference was not statistically significant (*p*-value: 0.953).

After 8 weeks of treatment, the ST + NHE group's mean HAM-D score decreased from 24.08 to 7.0 significantly (*p*-value: 0.002), the mean BECK-D score decreased from 25.38 to 12.08 significantly (*p*-value: 0.005), the mean FAB score increased from 16 to 17.75 significantly (*p*-value: 0.017), and the mean TAS score decreased from 61.31 to 52.50 after treatment, but the difference was not statistically significant (*p*-value: 0.099).

To compare treatment groups with each other, group differences are analyzed and only the FAB score is near the statistical significance (*p*-value: 0.06); other results were unsignificant.

### Comparative Results of Treatment Groups in Terms of Brain Networks With rs-fMRI

We did not find any network change by 32 × 32 and 32 × 167 ROI–ROI analysis, and we found three network changes and various inter-regional RSFC changes by 165 × 165 ROI–ROI analysis. They are the following:

#### The ST Group's RSFC Changes After Treatment

**Figure 1:**
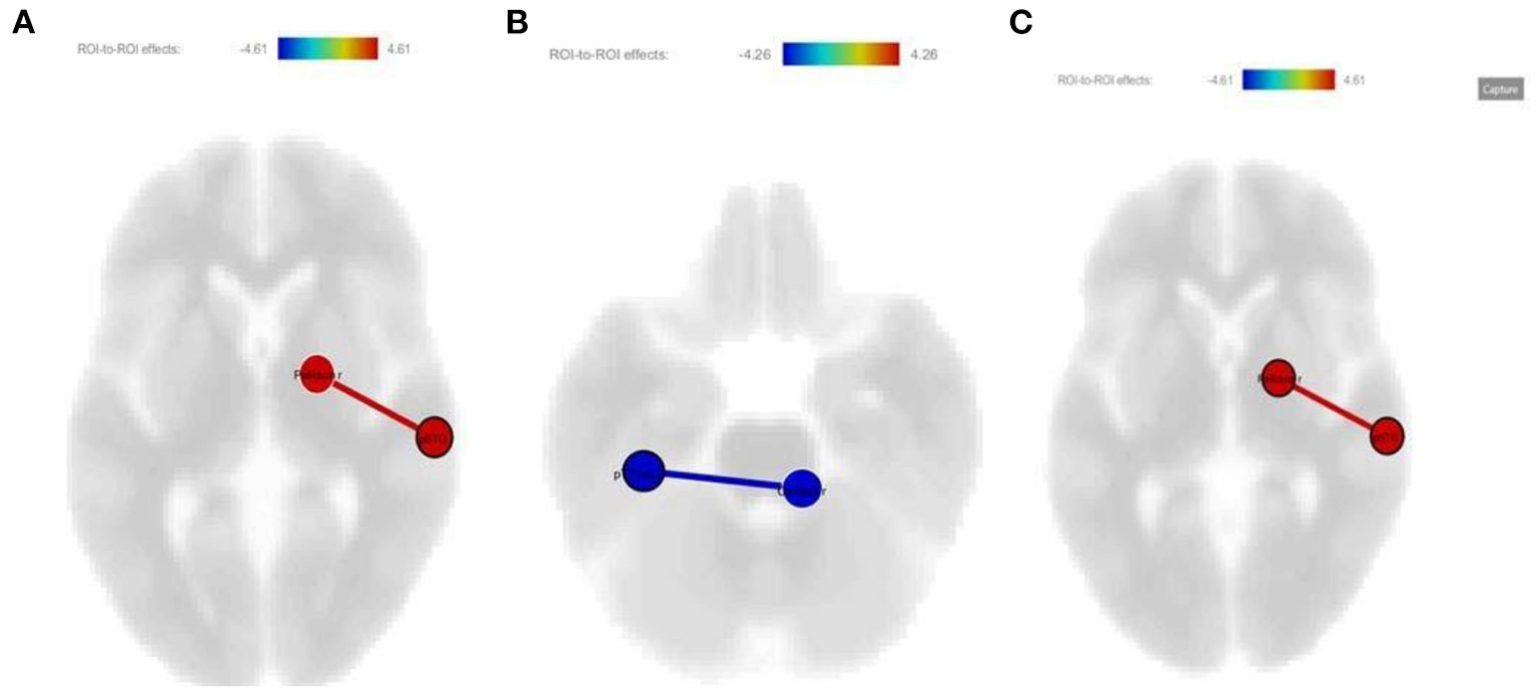
**(A)** Increased connectivity between right posterior STG and right pallidum after treatment in the medication-only group (*p-*unc: 0.0001; *p*-FDR: 0.0178); **(B)** Decreased connectivity between the left pTFus C and right cerebellum 3 after treatment treatment in the medication-only group (*p*-unc: 0.0002; *p*-FDR: 0.0385); **(C)** All ROI's are shown together as a picture.

The ST group started with 13 patients but concluded with 10 patients. When we compare post-treatment FC changes with pre-treatment within the group, the connectivity between the right posterior superior temporal gyrus (STG) and the right pallidum has increased mutually (*p*-unc: 0.0001; *p*-FDR: 0.0178) (Figure 1A), while the connectivity between the left posterior temporal fusiform cortex (pTFus C) and the right cerebellum-3 decreased mutually (*p*-unc: 0.0002; *p*-FDR: 0.0385) (Figure 1B). Additionally, all RSFC changes in the ST group are shown together as a picture (Figure 1C).

#### The ST + NHE Group's RSFC Changes After Treatment

**Figure 2:**
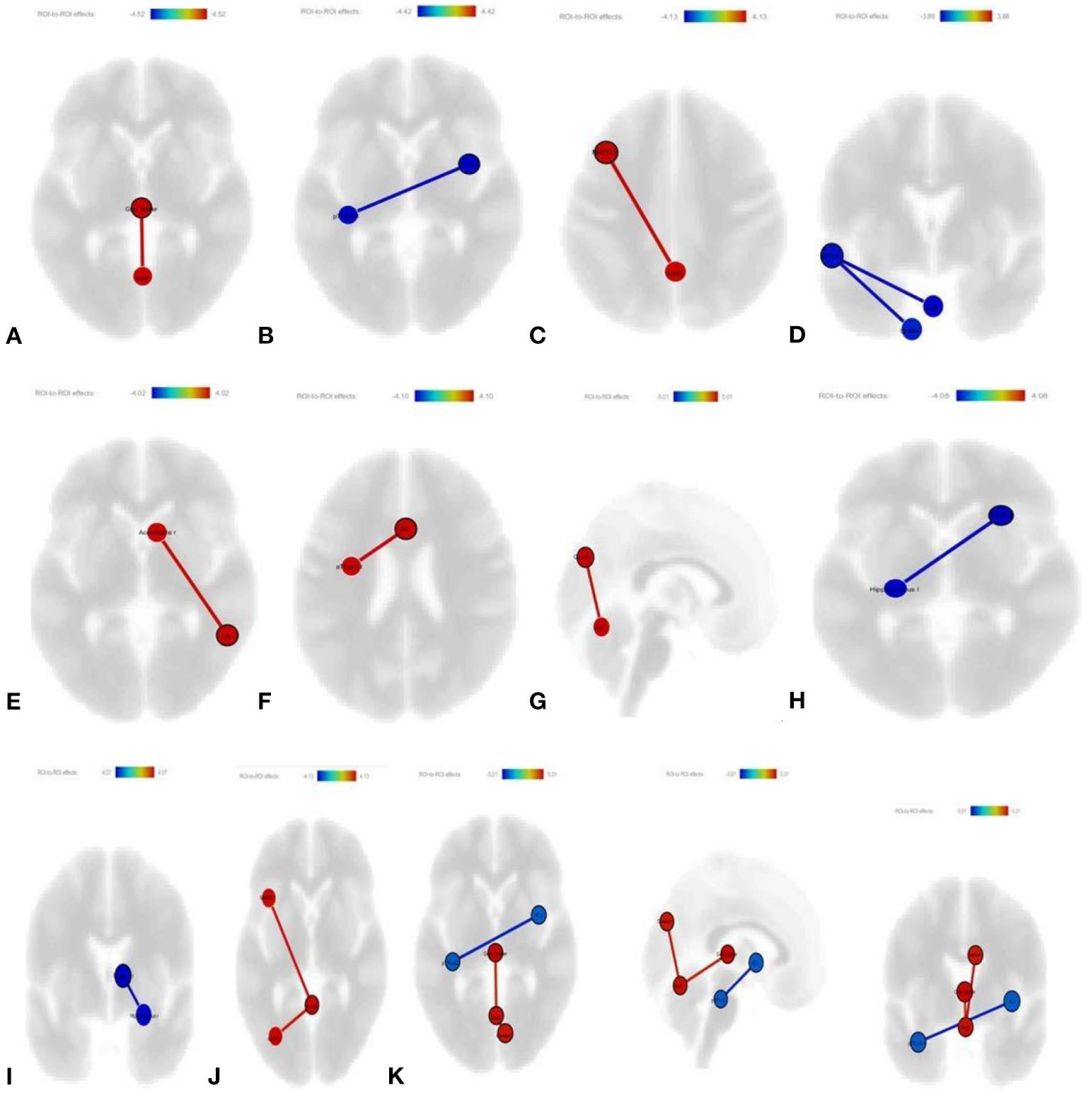
**(A)** Increased connectivity between deep gray matter and vermis 6 (*p-*unc: 0.0001; *p-*FDR: 0.0138); **(B)** decreased connectivity between right IC and left p TFus C (*p-*unc: 0.0002; *p-*FDR: 0.0177); **(C)** increased connectivity between vermis 4–5 and left MFG; **(D)** decreased connectivity between left anterior STG with vermis 8 (*p-*unc: 0.0004; *p-*FDR: 0.0333) and left cerebellum 9 (*p-*unc: 0.0004; *p-*FDR: 0.0333); **(E)** increased connectivity between right AG and right Nacc (*p-*unc: 0.0003; *p*-FDR: 0.0474); **(F)** increased connectivity between left anterior TFus C with ACC (*p-*unc: 0.0002; *p-*FDR: 0.0388); **(G)** increased connectivity between the cuneal right and vermis 6 (*p-*unc: 0.001; *p-*FDR: 0.042), **(H)** decreased connectivity between the right frontal orbital cortex and the left hippocampus (*p-*unc: 0.0002; *p-*FDR: 0.0404); **(I)** decreased connectivity between the right thalamus and the right hippocampus (*p-*unc: 0.0003; *p-*FDR: 0.0415); **(J)** increased connectivity between vermis 4–5 with left MFG (*p-*unc: 0.0002;*p-*FDR: 0.0346) and left superior LOC) (*p-*unc: 0.0004; *p-*FDR: 0.0346); **(K)** all ROI's are shown together as a picture.

The ST + NHE group started with 13 patients, but only 12 patients completed the study. Comparing post-treatment FC with pre-treatment status in the ST + NHE group, the connectivity between the deep gray matter and vermis-6 increased (*p*-unc: 0.0001; *p*-FDR: 0.0138) (Figure 2A); the connectivity between the right insular cortex (IC) and left pTFus C decreased (*p*-unc: 0.0002; *p*-FDR: 0.0177) (Figure 2B); the connectivity between the left middle frontal gyrus (MFG) and vermis-4–5 increased (*p*-unc: 0.0002; *p*-FDR: 0.0362) (Figure 2C); the connectivity between the left anterior STG and vermis-8 (*p*-unc: 0.0004; *p*-FDR: 0.0333) and left cerebellum-9 (*p*-unc: 0.0004; *p*-FDR: 0.0333) decreased (Figure 2D); the connectivity between the right angular gyrus (AG) and right nucleus accumbens (Nacc) increased (*p*-unc: 0.0003; *p*-FDR: 0.0474) (Figure 2E); the connectivity between the ACC and left anterior TFus C increased (*p*-unc: 0.0002; *p*-FDR: 0.0388) (Figure 2F); the connectivity between the right cuneus and vermis-6 increased (*p*-unc: 0.001; *p*-FDR: 0.042) (Figure 2G); the connectivity between the right orbitofrontal cortex (OFC) and left hippocampus decreased (*p*-unc: 0.0002; *p*-FDR: 0.0404) (Figure 2H); the connectivity between the right thalamus and right hippocampus decreased (*p*-unc: 0.0003; *p*-FDR: 0.0415) (Figure 2I); and the connectivity between the vermis-4–5 and left MFG (*p*-unc: 0.0002; *p*-FDR: 0.0346) and left superior lateral occipital cortex (LOC) (*p*-unc: 0.0004; *p*-FDR: 0.0346) (Figure 2J) increased mutually and significantly. Additonally, all ROI changes in ST + NHE are shown together as a picture (Figure 2K).

#### RSFC Comparison of Two Treatment Groups After the Treatment

**Figure 3:**
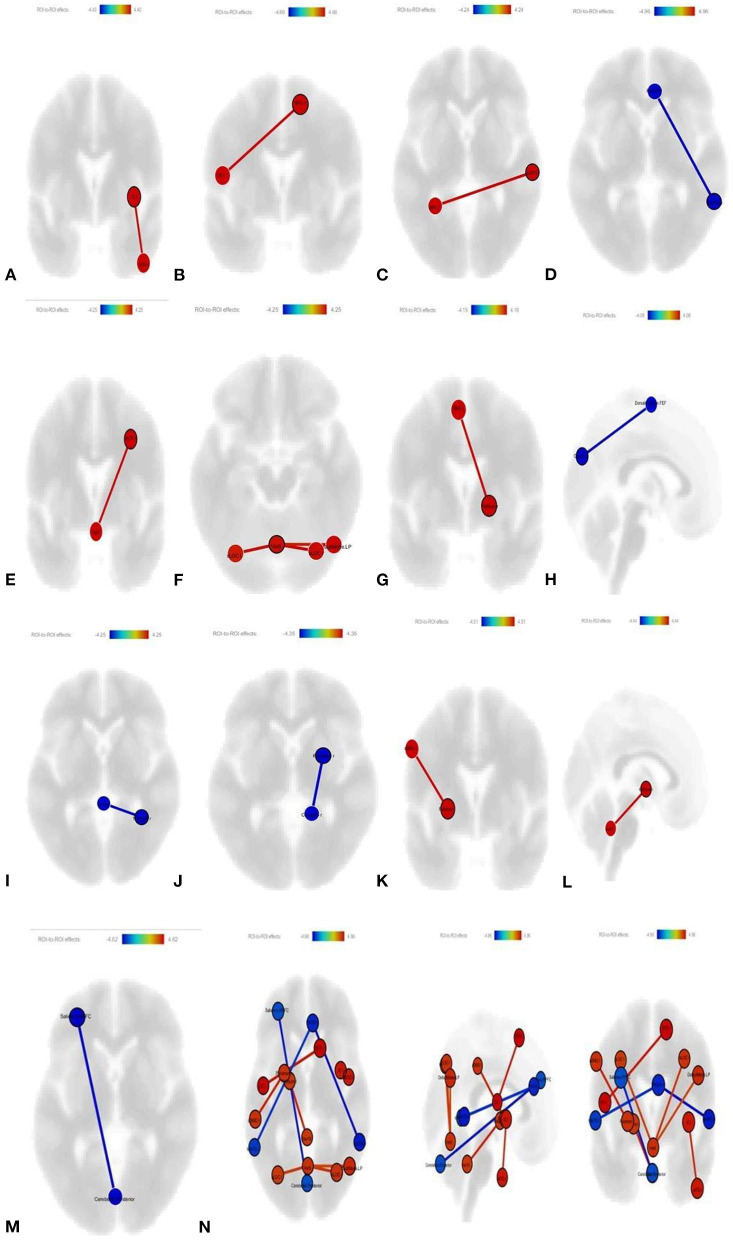
**(A)** Increased connectivity between right IC and right anterior ITG increased (*p-*unc: 0.0003; *p-*FDR: 0.224); **(B)** increased connectivity between right superior frontal gyrus (SFG) and the left COC (*p-*unc: 0.0002; *p-* FDR: 0.0125); **(C)** increased connectivity between right posterior STG and left SPL (*p-*unc: 0.0002; p-FDR: 0.0329); **(D)** decreased connectivity between right and left MTG temporooccipital part and right parasingulate gyrus (*p-*unc: 0.0003; *p-*FDR: 0.062); **(E)** increased connectivity between right superior LOC and vermis 6 (*p-*unc: 0.0003; *p-* FDR: 0.0319); **(F)** increased connectivity between vermis 6 and both right-left superior LOC (*p-*unc: 0.0006; *p-* FDR: 0.0245) and DMN LP (*p-*unc: 0.0009; *p-*FDR: 0.0245); **(G)** increased connectivity between the left SMA and the right pallidum (*p-*unc: 0.0002; *p-*FDR: 0.0366); **(H)** decreased connectivity between left cuneus and DAN FEF (*p-*unc: 0.0003 p FDR: 0.0476); **(I)** decreased connectivity between vermis 1–2 with right ToFus (*p-* unc: 0.0002 *p-*FDR: 0.0321); **(J)** decreased connectivity between putamen right and right cerebellum 4-5 (*p-*unc: 0.0002; p: FDR: 0.0254); **(K)** increased connectivity between putamen left and left anterior SMG (*p-*unc: 0.0001; *p-*FDR: 0.0174); **(L)** increased connectivity between the left pallidum and vermis 10 (*p-*unc: 0.0002; *p-*FDR: 0.0205); **(M)** decreased connectivity between the posterior cerebellum with left SN RPFC (*p-*unc: 0.0001; *p-* FDR:0.0137); **(N)** all ROI's are shown together.

Post-treatment, compared to the ST group, in the ST + NHE group, the connectivity between the right IC and right anterior ITG increased mutually (*p*-unc: 0.0003; *p*-FDR: 0.224) (Figure 3A); the connectivity between the right superior frontal gyrus (SFG) and the left central opercular cortex (COC) increased mutually (*p*-unc: 0.0002; *p*-FDR: 0.0125) (Figure 3B); the connectivity between the right posterior STG and left superior parietal lobule (SPL) increased mutually (*p*-unc: 0.0002; *p*-FDR: 0.0329) (Figure 3C); the connectivity between the right and left middle temporal gyrus (MTG) temporooccipital part and right parasingulate gyrus is mutually decreased (*p*-unc: 0.0003; *p*-FDR: 0.062) (Figure 3D); the connectivity between the right superior LOC and vermis-6 increased (*p*-unc: 0.0003; *p*-FDR: 0.0319) (Figure 3E); the connectivity between the vermis-6 and both right-left superior LOC (*p*-unc: 0.0006; *p*-FDR: 0.0245) and DMN lateral parietal (DMN LP) (*p*-unc: 0.0009; *p*-FDR: 0.0245) increased (Figure 3F); the connectivity between the left sumplementar motor area (SMA) and the right pallidum increased mutually (*p*-unc: 0.0002; *p*-FDR: 0.0366) (Figure 3G); the connectivity between the left cuneus and DAN frontal eye field (FEF) decreased mutually (*p*-unc: 0.0003 *p*-FDR: 0.0476) (Figure 3H); the interconnectivity between the vermis-1–2 and right temporal occipital fusiform cortex (ToFus) decreased mutually (*p*-unc: 0.0002; *p*-FDR: 0.0321) (Figure 3I); the connectivity between the right putamen and right cerebellum-4–5 decreased mutually (*p*-unc: 0.0002; *p*-FDR: 0.0254) (Figure 3J); the mutual relationship between the left putamen and left anterior supramarginal gyrus (SMG) increased (*p*-unc: 0.0001; *p*-FDR: 0.0174) (Figure 3K); the connectivity between the left pallidum and vermis-10 increased mutually (*p*-unc: 0.0002; *p*-FDR: 0.0205) (Figure 3L); and the connectivity between the posterior cerebellum and left SN rostral prefrontal cortex (RPFC) also decreased mutually (*p*-unc: 0.0001; *p*-FDR:0.0137) (Figure 3M). All ROI changes are shown as a picture (Figure 3N).

## Discussion

### Interpretation of Group Differences According to Sociodemographic Data and Neuropsychiatric Evaluation Scales

To support our hypothesis that NHE has an EMDR-like effect, we used FAB and TAS along with depression rating scales, because EMDR studies show improvement both in alexithymia and cognitive functions in different patient groups.

When we compare treatment groups with each other, both groups show significant improvement in BECK-D, HAM-D, and FAB tests. When treatment groups are compared with each other, only the FAB score difference is very near to the statistically significant limit (*p*-value: 0.06). The following discussed frontal RSFC changes in the ST + NHE group seem to explain that group difference. Although the *p*-value is not in the significance limit, it also has attracted that ST + NHE groups' TAS score *p*-value is 10 times smaller than the ST groups' *p*-value. Despite the significant group difference, why the result are not statistically significant may be due to the small sample size.

### Interpretation of Treatment Groups' RSFC Changes

If we evaluate the network changes in the context of depressive symptoms, we observed more regional connectivity changes in the ST + NHE group compared to the ST group, besides 13 network changes in the ST + NHE group compared to the ST group as group difference.

#### Interpretation of the ST Group's Post-treatment RSFC Changes

We observed increased connectivity between the right posterior STG and the right pallidum. Studies show that STG ReHo value increased in MDD and decreased after treatment; besides, STG is supposed to be a therapeutic target in MDD (20, 21). Low RSFC of the pallidum is suggested to be a biomarker related to limbic disorder in MDD (22). So, our result seems compatible with the current literature besides the first study investigating STG–pallidum connectivity in MDD.

Another result is decreased connectivity between the left pTFus C and right cerebellum-3. Cerebellum-3 is responsible for psychomotor activity (23). Cerebellar FC irregularities are emphasized in MDD pathology, but studies show opposite results (6, 24). Increased fusiform connectivity in MDD compared to healthy controls decreased after treatment. When treatment responses are compared, TR patients show lower fusiform connectivity (25). Besides, high suicide risk was found to be associated with low RSFC between the left fusiform and many regions (26). When we synthesize the fusiform's task (visual recognition and attention-memory functions) with cerebellar tasks together, we can interpret that their increased connectivity may be associated with improved attention and psychomotor activities (probably visual) post-treatment. This could be investigated with more detailed neuropsychological tests in the future.

So in general, the limbic–temporal region's connectivity increased while the cerebellum–fusiform's connectivity decreased in the ST group.

#### Interpretation of the ST + NHE Group's Post-treatment RSFC Changes

Since the NHE's effect on depression has not been proven yet, we interpreted RSFC changes mainly through connectivity changes associated with MDD. We evaluated the accuracy of our hypothesis *via* both neuropsychiatric tests and RSFC changes.

Our first result is post-treatment increased connectivity between the deep gray matter and vermis-6. Vermis-6 is called the oculomotor cortex and associated with hand–eye coordination and executive cognitive functions (27, 28). It was shown that FC between the PCC and vermis is associated with depression severity (29). Similar to our study results, the low amplitude of low-frequency fluctuations (ALFF) in the gray matter of MDD patients compared to healthy controls increased after antidepressant treatment (30). A treatment-response comparison study in MDD shows increased connectivity among the vmPFC and vermis in treatment responders compared to non-responders (31). Our result is new investigating the FC between the gray matter and vermis-6 in MDD.

Another result is decreased connectivity between the right IC and left pTFus C. Insular connectivity changes in MDD give contradictory results (32, 33). A study showed a positive correlation between the severity of hopelessness in MDD and right ventral ACC and right insular ReHo (34). MDD patients who attempted suicide showed high RSFC between the left amygdala and the right insula, unlike MDD patients who did not attempt suicide (35). The right insula has been reported to play a role in empathy and emotional regulation and attention to internal body functions and rumination (36, 37). The fusiform also plays a role in neutral facial expression recognition and emotion expression. Fusiform ALFF abnormalities have been shown to indicate impairments in social and emotion-regulation circuits. From this point of view, post-treatment decreased connectivity between the insula and fusiform may be the underlying cause of treatment responses such as negative perception of environmental stimuli (facial expressions), decreased inward tendency of thought, increased attention to the outside, and more objective evaluation of stimuli. This change seen only in the ST + NHE group gives us hope that NHE can regulate the negative perception like the EMDR.

Another result is increased connectivity between the left MFG and vermis-4–5. The vermis-4–5 is mainly associated with motor functions, but the role of the cerebellum in regulating attention, cognition, and emotion has recently been considered increasingly (38). The MFG is one of the two main regions of the cognitive control network (CCN), which is thought to be responsible for high cognitive functions (39). Compared to healthy individuals, the ReHo value in the MDD was low in the left hemisphere, primarily in the left MFG (15). A significant decrease in the left MFG ALFF values is suggested to evaluate potential suicide risk in MDD (40). Our result of increased connectivity in the left MFG seems compatible with the literature. This can be considered as a causal indicator of improvement in cognitive functions after antidepressant treatment and can explain FAB score impairment.

Another result is decreased connectivity from the left anterior STG to vermis-8 and left cerebellum-9. Increased STG ReHo value is reported in MDD (15). Increased RSFC in the left cerebellum has been considered important for suicide risk in MDD (41). The decreased connectivity between the left cerebellum and left anterior STG after treatment from the same study seems to be compatible with our study result.

Another result is increased connectivity between the right AG and the right Nacc. TR patients have a low FC between the right Nacc and left SFG and right precuneus and have high FC between the right Nacc and right cingulate gyrus and MFG compared to treatment responders in MDD treatment (42). Our study is compatible with this study. Based on the knowledge that the Nacc is the most important component of the reward circuit, we can interpret that the NHE may activate the reward system, as well.

Another significant FC change is increased connectivity between the right cuneus and vermis-6. Compatible with our result, in a treatment-response fMRI study, TR patients showed less connectivity in the left cuneus compared to treatment responders (43).

We found increased connectivity between the ACC and left anterior TFus C. Compatible with our study, an MDD study conducted in adolescents reported a reduced RSFC especially in the subgenual anterior cingulate cortex (sgACC) (44). Contrary results like decreased ACC connectivity after treatment and high FC in treatment resistance are available in the current literature (43). The regulatory effect of the ACC on mood-regulating limbic areas has been known well and low connectivity means inability in emotion regulation. The TFus plays a role in understanding facial expression and regulating the emotional response to negative stimuli. So, increased connectivity between the TFus and ACC seems to contribute to emotion regulation and improvement in TAS score.

Decreased connectivity between the OFC and the left hippocampus is another result. The OFC plays an important role in social and emotional adaptive and goal-oriented behaviors and functions as a component of central executive network (CEN) and DMN (45). A very recent study suggested that OFC activity may be a complementary response to reduce negative emotional responses. Adolescents who experience emotional and behavioral irregularities compared to their peers showed high FC and low volume in the right OFC during emotional control and self-inhibition (46). Connections of the hippocampus with the PFC, thalamus, amygdala, basal ganglia, and hypothalamus mediate emotion regulation *via* the corticolimbystritotalamic emotion regulation circuit (CERC) (47). The importance of hippocampal regulation was emphasized in the treatment of MDD, including animal studies (48). Altogether, we can interpret that decreased connectivity between them may decrease negative affect and increase emotion regulation ability.

We also found decreased connectivity between the right thalamus and the right hippocampus. Thalamic connectivity changes in MDD are very common but have contradictory results (49, 50). The thalamus and hippocampus play a role in the CERC. We found nearly statistically significant TAS score group difference in the ST + NHE group, which can be interpreted as an improved ability to understand emotions more accurately *via* regulated CERC. We think that the relationship between these regions may be related to a negative bias in depression, such as past negative memories coming to mind; therefore, decreased FC may regulate negative bias.

Increased connectivity between the vermis-4–5 and left superior LOC is the last result. The vermis-4–5 is associated with motor functions, so increased connectivity with several regions can be interpreted as an expected result, because depression causes psychomotor inhibition in general. Projections from the LOC to temporal-parietal areas play a role in sensing objects' movement. Decreased occipital connectivity with many brain regions is shown in MDD (51). So, post-treatment increased connectivity between the LOC and vermis-4–5 seems compatible with the literature. The relationship between the LOC and vermis may be related to visual and psychomotor improvements of depression in remission *via* NHE.

Altogether, the connectivity of some parts of the temporal and parietal gyrus and the cerebellum gives opposite results with different regions, while the connectivity of some parts of the frontal and occipital cortex increased. We observed more significant network changes in the ST + NHE group, and more of them seem to be related to emotion and attention regulation circuits, which can support more improvement in TAS and FAB scores. Increased ACC connectivity, which also draws the most attention in EMDR studies, seems to support our hypothesis that NHE may have EMDR-like effect. Similar to the EMDR method, negative emotional bias decreased due to significant connectivity changes in the limbic, temporal, and reward system, and by objective evaluation of internal and environmental stimuli, the TAS score was found more decreased (nearly statistically significant) in the ST + NHE group compared to the ST group in addition to other improved depression scales.

### Comparison of RSFC Changes of Two Different Treatment Groups and the Potential Effect of NHE on Treatment

A comparison between two treatment groups constitutes the most important part in showing the effect of NHE on treatment. Since NHE is an unproven method yet, we highlighted our results consistent with FC changes found in the literature regarding depression and its treatment. Otherwise, we would just assess the circuits that are related to achieving the habit of NHE as a result of the treatment.

Post-treatment RSFC changes in the ST + NHE group compared to the ST group as group differences are evaluated here. That results can be evaluated as NHE's effect on MDD, supported by the difference in neuropsychiatric test scores between groups.

Increased connectivity between the right insular cortex and the right anterior ITG is our first result. Compatible with our result, decreased ITG connectivity in MDD and a positive correlation between low ITG connectivity and symptom severity are reported in the literature (52).

Increased connectivity between the right SFG and left COC is another result. Post-treatment increased activity conforms to our expectations based on the available literature showing low frontal functions in MDD (53). The SFG plays an important role in working memory. Increased FC between these regions, both located in the frontal lobe, indicates an improvement in cognitive functions compatible with more FAB score impairment in the ST + NHE group. NHE has shown hope for reversing executive dysfunctions in depression.

Another result is increased connectivity between the right posterior STG and the left SPL. In first-episode MDD patients, the internal activity of the right ITG, STG, and left MTG increased significantly compared to those who entered remission and healthy controls (21). So, increased ITG FC seems inconsistent with the literature. The SPL is involved in attention shifting/changing function (54), but its FC in MDD is not studied yet.

Another group difference is decreased connectivity between the right and left MTG temporooccipital part and the right parasingulate gyrus. We see studies in line with our results reporting increased MTG FC in MDD (21, 55). Functionally, the parasingulate is known to be involved in the mentalization ability and self-monitorization. The parasingulate works as default and is important in self-knowledge, inner thought process, and social mind (56). The MTG is included in DMN, and its high activity is related to behavior problems in MDD (57). According to the functions of these regions, their abnormal connectivity is associated with increased inner focus and rumination and decreased social awareness and relationships, which are very common symptoms of depression. So, increased FC of these regions seems as the underlying result of decreased depressive symptoms *via* NHE.

Another group difference is increased connectivity between the vermis-6 and right-left superior LOC and between vermis-6 and DMN LP. Increased connectivity between the LOC, which is responsible for visual perception, and vermis-6, which is responsible for hand–eye coordination, may be related to improved visual and motor coordination *via* NHE. Increased DMN connectivity and normalization after antidepressant treatment in MDD has been demonstrated in many studies (58). With the knowledge that both the DMN LP is involved in cognitive functions such as attention to the external environment and the vermis-6 plays a role in cognitive functions, we can argue that post-treatment increased connectivity between DMN LP and vermis-6 may be the underlying cause of improvement in attention and cognitive functions. In our study, compatible with RSFC changes, we achieved nearly significant FAB improvement in the ST + NHE group, but additional neuropsychological tests would be needed for more detailed cognitive assessment.

Increased connectivity between the left SMA and the right pallidum is another result. Low connectivity of the right pallidum in MDD was associated with emotional and reward circuit regulation problems *via* corticostriatopallidothalamolimbic circuits. So, increased pallidum connectivity is compatible with the literature. The most important functions of the pallidum, which is included in SMA, sensory motor network (SMN), and CEN, are to ensure balance in motor activities, motion control, impulsivity, and inhibition control. A meta-analysis reported decreased volume besides increased RSFC of the right SMA, and it was stated to be related to psychomotor retardation in the first-episode MDD patients (59). In contrast to this data, we found an increase in the left SMA FC after treatment, which should be caused by the other seed region or different study design. Increased connectivity between the SMA and pallidum after treatment may be related to the decrease in emotional impulsivity or remission of psychomotor retardation or agitation in our study, but we cannot make a clear interpretation since we did not separately examine sub-symptoms of depression.

Another significant group difference is decreased connectivity between the left cuneus and DAN FEF. We can state that our research results are compatible with the current studies we have mentioned above showing that the cuneus connectivity is higher in MDD patients than healthy controls. The cuneus takes to play in visual processing, while DAN FEF takes part in visual attention orientation and working memory. There is no clear information about whether connectivity changes between the cuneus and DAN FEF have a role in depressive symptomatology. Their increased connectivity could be related to the especially visual part of attention, but we did not make a neuropsychiatric test to prove it. This result may have emerged as a group difference caused by NHE only as an increase in hand–eye coordination and could not be related to any depressive symptom.

Another result is the decreased connectivity between the right ToFus and vermis-1–2. The ToFus is a part of the fusiform that lies between the occipital and temporal lobes. The vermis-1–2 is generally held responsible for motor motion balance and posture. In literature, FC of the vermis generally increases after MDD treatment, but we could not find any data specifically about the vermis-1–2. We can only interpret that post-treatment increased connectivity between them could be related to increased visual attention and psychomotor activity, as well.

Another result is decreased connectivity between the right putamen and right cerebellum-4–5, besides increased connectivity between the left putamen and the left anterior SMG. Low connectivity of the putamen is reported to cause dysfunctions in reward and emotion processing circuits in MDD (47). Another study reports decreased connectivity between the putamen and the hypothalamus after 8 weeks of antidepressant treatment (43). A similar result like ours showed low ALFF in the right putamen in MDD patients with remission (60). Like the cerebellum, the putamen has a role in motor motion regulations. Decreased connectivity between the putamen and the cerebellum may explain the improvement of psychomotor retardation seen in MDD. The SMG is a region included in CCN and SN and is held responsible for high cognitive functions. MDD patients show high SMG RSFC that decreases in those who go into remission (58). The connectivity between the MPFC component of the DMN and the SMG component of the left SN was found to be significantly higher in the MDD group than the healthy controls, which seems associated with increased rumination and increased cognitive processing, albeit in a negative way. In our previous study, where we compared MDD patients with healthy controls, we detected decreased SMG RSFC in the MDD group, which is consistent with our current study results. Based on the knowledge that low SMG connectivity reflects the difficulty of the transition between targeted attention and emotional response, we can argue that increased connectivity between the SMG and putamen after treatment is the causal factor that mediates the improvement of attention and learning processes that are disturbed in MDD.

Another significant group difference is increased connectivity between the left pallidum and vermis-10 in the ST + NHE group. The vermis-10 is responsible for balance and eye movements. In the literature, we could not find any connectivity study about the vermis-10 in MDD. We have mentioned extensively the increased pallidum's connectivity while interpreting other results and stated that it plays a role in processes such as motor movement balance and inhibition of impulsivity. In terms of their functions, increased connectivity between them may be related to the improvement in psychomotor activation or retardation seen in MDD.

The final difference is decreased connectivity between the left SN RPFC and the posterior cerebellum. Contrary to our results, a meta-analysis showed that the cerebellum's intrinsic activity decreased in the MDD subgroup that did not take any treatment, compared to the treatment group (61). In our previous study, where we compared the MDD group with healthy controls, we found increased RPFC connectivity in MDD, which seems consistent with our current study result. RPFC plays a role in paying attention to environmental stimuli and inner thoughts. The cerebellum has been shown to play a key role in emotional perception and cognitive processing in mood disorders (62). The posterior part of the cerebellum is responsible for these non-motor functions and is associated with CCN, SN, and DMN. It is shown that the cerebellum's relationship with the prefrontal region plays a role in the pathophysiology of MDD (63). Post-treatment decreased connectivity between these regions may be related to the directing of attention to external stimuli rather than internal stimuli; thus, environmental attention increases and internal ruminative thoughts decrease. It has long been known that the limbic system (SN here) provides the integration of experiences originating from outside and inside the organism.

Compared to the ST group, the ST + NHE group showed both significantly high improvement in depression/neuropsychiatric scales and much more connectivity changes. Some regions' connectivity may change their function only because of the NHE of course, but accompanying clinic improvement supports our hypothesis that depressive symptoms improved more in the ST + NHE group (especially in attention, motor function, and emotional pathways) through the NHE like the EMDR effect. By shifting the high cortical and limbic firing to the fusiform and visual cortex during the explanation of autobiographical memory after EMDR therapy, decreased corticolimbic hyperactivity appears to be the most prominent FC change (64). According to the emotional asymmetry theory, it has been suggested that while the right hemisphere dominates the left hemisphere during emotional expression and perception, increased left dominance after successful EMDR means cognitive processing of stressful memories (65). It is known that in MDD, top-down control from the DLPFC and ACC to the limbic system is impaired compared to healthy controls, and this causes increased emotional responses. It has been shown that increased connectivity between the amygdala and ACC increases the recall of past negative and/or traumatic memories, flashbacks, and ruminative thinking, and the symptoms regress with the decrease in that connectivity after EMDR (66). Compatible with the EMDR effect, we obtained increased frontal and occipital connectivities that contribute to improvement in neuropsychiatric scales. In the NHE group, we obtained various limbic and temporal fusiform network changes as seen in EMDR studies. We would like to emphasize that with the activation of similar visual pathways during NHE, the shift of limbic hyper activation to the fusiform, occipital visual fields result in a network change similar to EMDR as we hypothesized. We think that more information should be investigated by comparing the effectiveness of NHE and EMDR in a treatment-response comparison study.

### Limitations and Recommendations

Comparing treatment results of an unproven method (NHE) is an important limitation of our study. By including a healthy control group as a pre-study or as part of this study, comparing rs-fMRI changes before and after NHE, it would be methodologically safer to identify network changes that may be due to NHE alone. However, this study is also an important step in the path to evidence because brain imaging is one of the best ways to provide the effect of a new method.

One of the major limitations of rs-fMRI is that the method cannot measure directly the neuronal activity but only the cerebral blood flow changes associated with it. Blood volume, basal blood oxygenation, basal metabolic rate, basal flow, basal blood carbon dioxide level, blood pressure, hematocrit, and the level of neuronal activity are physiological factors that affect BOLD. So, there is a need for correction of physiological data due to respiratory and cardiac activity. Due to its *post hoc* analysis, the ICA method is better equipped than the ROI analysis method to extract physiological noise. Thus, our study is subject to the limitations of the ROI analysis method.

As rs-fMRI is an emerging field, standards for data collection and processing vary widely between studies, so we see incoherent results in the literature, most probably caused by using different methods. Our study was limited to the seed ROIs selected by the analysis program, as the ROI–ROI method was preferred. Therefore, while some networks and anatomical regions are examined in more detail than others, we may have ignored some regions studied in more detail by different analysis methods.

MDD often varies considerably in symptomatology; comorbidity is not rare. It is more difficult to establish a clear relationship between MDD patients with comorbidity and rs-fMRI changes. In the literature, MDD patients who responded or did not respond to treatment and similarly patients with first or recurrent attacks show different rs-fMRI changes. When these details are taken into attention, our study sample is not homogenous enough. Besides, we chose right-handed (left hemisphere-dominant) subjects to provide radiologic uniformity, but this is another shortcoming of our study, because cortical dominance may be different from hand dominance, albeit rarely.

RSFC changes may be associated with different disorders; however, it is not yet understood in what way it causes which disorder (for example, RSFC increases between DMN and SN in both MDD and schizophrenia). Although the need for correlation studies between symptoms and RSFC changes is obvious, there are very few studies in the current literature. In our study, we evaluated attention processes only with FAB and did not evaluate depression sub-symptoms (psychomotor movement, sleep, etc.) with separate specific scales. It has caused us to be limited to interpretive explanation only. In future studies, evaluating each symptom specifically in terms of specific scales and RSFC in terms of correlation will provide clearer data. Finally, before conducting a follow-up study for possible relapses, it is also hard to claim a beneficial effect of NHE on the long view.

### Conclusion and Recommendations

Despite all pharmacological treatment approaches, incomplete remission and high relapse rates remain for many patients with MDD. Physicians refer MDD patients, who do not have an adequate and permanent response to drug therapy or who are not suitable to use medications, to psychosomatic treatment methods or psychotherapies. However, psychotherapy options cannot be used as often as drugs in the treatment of depression in our country because of various limitations. We claim that the NHE, which can be evaluated as a self-administered therapy method at home, can decrease time and cost spent on drugs and psychotherapies. We showed in our small group that NHE has a positive contribution to MDD treatment like the EMDR effect.

In the rising trend, RSFC studies promise developments in elucidating the complex pathophysiology of MDD. Our effort to evaluate all brain regions and networks that may be associated with MDD, without being limited to a few regions like the first studies in this field, will contribute to the current literature.

Problems that draw attention in RSFC studies are the following. Studies examining network changes show great differences between both methods and nomenclature. Future studies should be carried out with larger samples, and a common strategy for data acquisition and analysis should be generated. A specific region with different regions can yield opposite results, but increased connectivity indicates regions giving positive signals at the same time, while decreased connectivity indicates regions giving negative signals simultaneously. One possible reason why the same region gives opposite results in the same person may be that the area it is associated with is strongly influenced by or the analysis method takes left-right-anterior-posterior parts of the same region into account for a precise distinction. Similarly, a recently noticed situation in structural imaging is that a volume reduction can cause increased FC *via* a compensation mechanism, so increased connectivity does not always mean increased internal activity. Except for a single study, there is not any study investigating the relationship between RSFC with structural abnormalities and task-fMRI studies. Since we think it can illuminate that confusion in the literature, we recommend to carry out the structural/functional and rs-fMRI studies together.

## Data Availability Statement

The original contributions presented in the study are included in the article/supplementary material, further inquiries can be directed to the corresponding author.

## Ethics Statement

T.C. Bezmialem Vakıf University Clinical Research Ethics Committee approved all steps of the study by the number 3.2018/8. Informed consent about the study and publication circumstances was obtained from all participants.

## Author Contributions

All authors have made a substantial contribution to the conception, design, gathering, analysis, and/or interpretation of data, writing, intellectual content of the article, and read and approved the manuscript for submission to Frontiers in Psychiatry.

## Funding

This study was funded by T.C. Bezmialem Vakıf University BAP Commitee with the project approval code 3.2018/8.

## Conflict of Interest

The authors declare that the research was conducted in the absence of any commercial or financial relationships that could be construed as a potential conflict of interest.

## Publisher's Note

All claims expressed in this article are solely those of the authors and do not necessarily represent those of their affiliated organizations, or those of the publisher, the editors and the reviewers. Any product that may be evaluated in this article, or claim that may be made by its manufacturer, is not guaranteed or endorsed by the publisher.

## References

[1] Kenny ER O'brien JT Cousins DA Richardson J Thomas AJ Firbank MJ. Functional connectivity in late-life depression using resting-state functional magnetic resonance imaging. Am J Geriatr Psychiatry. (2010) 18:643–51. 10.1097/JGP.0b013e3181cabd0e 20220591

[2] Wang L Hermens D Hickie I Lagopoulos J. A systematic review of resting-state functional-MRI studies in major depression. J Affect Disord. (2012) 142:6–12. 10.1016/j.jad.2012.04.013 22858266

[3] Posner J Hellerstein DJ Gat I Mechling A Klahr K Wang Z. Antidepressants normalize the default mode network in patients with dysthymia. JAMA Psychiatry. (2013) 70:373–82. 10.1001/jamapsychiatry.2013.455 23389382 PMC3935731

[4] Wu QZ Li DM Kuang WH Zhang TJ Lui S Huang XQ. Abnormal regional spontaneous neural activity in treatment-refractory depression revealed by resting-state fMRI. Hum Brain Mapp. (2011) 32:1290–9. 10.1002/hbm.21108 20665717 PMC6870367

[5] Anand A Li Y Wang Y Gardner K Lowe MJ. Reciprocal effects of antidepressant treatment on activity and connectivity of the mood regulating circuit: an FMRI study. J Neuropsychiatry Clin Neurosci. (2007) 19:274–82. 10.1176/jnp.2007.19.3.274 17827412 PMC3465666

[6] Yang R Zhang H Wu X Yang J Ma M Gao Y. Hypothalamus-anchored resting brain network changes before and after sertraline treatment in major depression. Biomed Res.Int. (2014):2014. 10.1155/2014/915026 24772438 PMC3977500

[7] Disner SG Beevers CG Haigh EA Beck AT. Neural mechanisms of the cognitive model of depression. Nature Rev Neurosci. (2011) 12:467. 10.1038/nrn3027 21731066

[8] Bergmann U. EMDR's neurobiological mechanisms of action: A survey of 20 years of searching. J EMDR Pract Res. (2010) 4:22–42. 10.1891/1933-3196.4.1.22

[9] Rasolkhani-Kalhorn T Harper ML. EMDR and low frequency stimulation of the brain. Traumatology. (2006) 12:9–24. 10.1177/153476560601200102

[10] van der Kolk BA Spinazzola J Blaustein ME Hopper JW Hopper EK Korn DL. A randomized clinical trial of eye movement desensitization and reprocessing (EMDR), fluoxetine, and pill placebo in the treatment of posttraumatic stress disorder: treatment effects and long-term maintenance. J Clin Psychiatry. (2007) 68:37–46. 10.4088/JCP.v68n0105 17284128

[11] Hofmann A Hilgers A Lehnung M Liebermann P Ostacoli L Schneider W. Eye movement desensitization and reprocessing as an adjunctive treatment of unipolar depression: A controlled study. J EMDR Pract Res. (2014) 8:103–12. 10.1891/1933-3196.8.3.103

[12] Wood E Ricketts T Parry G. EMDR as a treatment for long-term depression: A feasibility study. Psychol Psychother. (2018) 91:63–78. 10.1111/papt.12145 28834138 PMC5836996

[13] Hall JE. Guyton y Hall. Elsevier Health Sciences Spain (2011). p. 745.

[14] Kaprinis G Nimatoudis J Karavatos A Kandylis D Kaprinis S. Functional brain organization in bipolar affective patients during manic phase and after recovery: a digit dichotic listening study. Percept Mot Skills. (1995) 80:1275–82. 10.2466/pms.1995.80.3c.1275 7478888

[15] Li M Xu H Lu S. Neural basis of depression related to a dominant right hemisphere: A resting-state fMRI study. Behav. Neurol. (2018) 2018. 10.1155/2018/5024520 29971137 PMC6008682

[16] Beck AT Ward CH Mendelson M Mock J Erbaugh J. An inventory for measuring depression. Arch Gen Psychiatry. (1961) 4:561–71. 10.1001/archpsyc.1961.01710120031004 13688369

[17] Akdemir A Örsel S Dag I Türkçapar H Işcan N Özbay H. Hamilton Depresyon Derecelendirme Ölçegi (HDDÖ)'nin geçerligi, güvenirligi ve klinikte kullanimi. Psikiyatri Psikoloji Psikofarmakoloji Dergisi. (1996) 4:251–9.

[18] Chapman LJ Chapman JP. The measurement of handedness. Brain Cogn. (1987) 6:175–83. 10.1016/0278-2626(87)90118-7 3593557

[19] Dubois B Slachevsky A Litvan I Pillon B. The FAB: a frontal assessment battery at bedside. Neurology. (2000) 55:1621–6. 10.1212/WNL.55.11.1621 11113214

[20] Lai C-H Wu Y-T. Frontal regional homogeneity increased and temporal regional homogeneity decreased after remission of first-episode drug-naive major depressive disorder with panic disorder patients under duloxetine therapy for 6 weeks. J Affect Disord. (2012) 136:453–8. 10.1016/j.jad.2011.11.004 22137181

[21] Yang C Zhang A Jia A Ma JX Sun N Wang Y. Identify abnormalities in resting-state brain function between first-episode, drug-naive major depressive disorder and remitted individuals: a 3-year retrospective study. Neuroreport. (2018) 29:907–16. 10.1097/WNR.0000000000001054 29912848

[22] Anand A Li Y Wang Y Wu J Gao S Bukhari L. Activity and connectivity of brain mood regulating circuit in depression: a functional magnetic resonance study. Biol Psychiatry. (2005) 57:1079–88. 10.1016/j.biopsych.2005.02.021 15866546

[23] Grodd W Hülsmann E Ackermann H. Functional MRI localizing in the cerebellum. Neurosurgery Clinics. (2005) 16:77–99. 10.1016/j.nec.2004.07.008 15561530

[24] Schraa-Tam CK Rietdijk WJ Verbeke WJ Dietvorst RC Van Den Berg WE Bagozzi RP. fMRI activities in the emotional cerebellum: a preference for negative stimuli and goal-directed behavior. The Cerebellum. (2012) 11:233–45. 10.1007/s12311-011-0301-2 21761197 PMC3311856

[25] Guo W-b Liu F Chen J-d Gao K Xue Z-m Xu X-j. Abnormal neural activity of brain regions in treatment-resistant and treatment-sensitive major depressive disorder: a resting-state fMRI study. J Psychiatr Res. (2012) 46:1366–73. 10.1016/j.jpsychires.2012.07.003 22835912

[26] Cao J. Chen J-m, Kuang L, Ai M, Fang W-d, Gan Y, et al. Abnormal regional homogeneity in young adult suicide attempters with no diagnosable psychiatric disorder: a resting state functional magnetic imaging study. Psychiatry Res: Neuroimaging. (2015) 231:95–102. 10.1016/j.pscychresns.2014.10.011 25496980

[27] Park IS Lee NJ Rhyu IJ. Roles of the Declive, Folium, and Tuber Cerebellar Vermian Lobules in Sportspeople. J Clini Neurol. (2018) 14:1–7. 10.3988/jcn.2018.14.1.1 29141275 PMC5765239

[28] Chen SA Desmond JE. Temporal dynamics of cerebro-cerebellar network recruitment during a cognitive task. Neuropsychologia. (2005) 43:1227–37. 10.1016/j.neuropsychologia.2004.12.015 15949507

[29] Alalade E Denny K Potter G Steffens D Wang L. Altered cerebellar-cerebral functional connectivity in geriatric depression. PLoS ONE. (2011) 6:e20035. 10.1371/journal.pone.0020035 21637831 PMC3102667

[30] Fang J Mao N Jiang X Li X Wang B Wang Q. Functional and anatomical brain abnormalities and effects of antidepressant in major depressive disorder: combined application of voxel-based morphometry and amplitude of frequency fluctuation in resting state. J Comput Assist Tomogr. (2015) 39:766–73. 10.1097/RCT.0000000000000264 26125296

[31] Emam H Steffens DC Pearlson G Wang L. Increased ventromedial prefrontal cortex activity and connectivity predict poor sertraline treatment outcome in late-life depression. Int J Geriatr Psychiatry. (2019). 10.1002/gps.5079 30761621 PMC6480406

[32] Cullen KR Gee DG Klimes-Dougan B Gabbay V Hulvershorn L Mueller BA. A preliminary study of functional connectivity in comorbid adolescent depression. Neurosci Lett. (2009) 460:227–31. 10.1016/j.neulet.2009.05.022 19446602 PMC2713606

[33] Tu Z Jia YY Wang T Qu H Pan JX Jie J. Modulatory interactions of resting-state brain functional connectivity in major depressive disorder. Neuropsychiatr Dis Treat. (2018) 14:2461. 10.2147/NDT.S165295 30319258 PMC6167995

[34] Yao Z Wang L Lu Q Liu H Teng G. Regional homogeneity in depression and its relationship with separate depressive symptom clusters: a resting-state fMRI study. J Affect Disord. (2009) 115:430–8. 10.1016/j.jad.2008.10.013 19007997

[35] Kang S-G Na K-S Choi J-W Kim J-H Son Y-D Lee YJ. Resting-state functional connectivity of the amygdala in suicide attempters with major depressive disorder. Prog Neuro-Psychoph Biol Psychiatry. (2017) 77:222–7. 10.1016/j.pnpbp.2017.04.029 28445688

[36] Gu X Hof PR Friston KJ Fan J. Anterior insular cortex and emotional awareness. J Comp Neurol. (2013) 521:3371–88. 10.1002/cne.23368 23749500 PMC3999437

[37] Fan Y Duncan NW de Greck M Northoff G. Is there a core neural network in empathy? An fMRI based quantitative meta-analysis. Neurosci & Biobehavioral Rev. (2011) 35:903–11. 10.1016/j.neubiorev.2010.10.009 20974173

[38] Stoodley CJ Schmahmann JD. Evidence for topographic organization in the cerebellum of motor control versus cognitive and affective processing. Cortex. (2010) 46:831–44. 10.1016/j.cortex.2009.11.008 20152963 PMC2873095

[39] Clasen PC Beevers CG Mumford JA Schnyer DM. Cognitive control network connectivity in adolescent women with and without a parental history of depression. Dev Cogn Neurosci. (2014) 7:13–22. 10.1016/j.dcn.2013.10.008 24270043 PMC4209722

[40] Cao J Chen X Chen J Ai M Gan Y Wang W. Resting-state functional MRI of abnormal baseline brain activity in young depressed patients with and without suicidal behavior. J Affect Disord. (2016) 205:252–63. 10.1016/j.jad.2016.07.002 27467529

[41] Zhang S. Chen J-m, Kuang L, Cao J, Zhang H, Ai M, et al. Association between abnormal default mode network activity and suicidality in depressed adolescents. BMC psychiatry. (2016) 16:337. 10.1186/s12888-016-1047-7 27688124 PMC5041526

[42] Hou Z Gong L Zhi M Yin Y Zhang Y Xie C. Distinctive pretreatment features of bilateral nucleus accumbens networks predict early response to antidepressants in major depressive disorder. Brain Imaging Behav. (2018) 1–11. 10.1007/s11682-017-9773-0 28971301

[43] Dichter GS Gibbs D Smoski MJ. A systematic review of relations between resting-state functional-MRI and treatment response in major depressive disorder. J Affect Disord. (2015) 172:8–17. 10.1016/j.jad.2014.09.028 25451389 PMC4375066

[44] Wu H Sun H Xu J Wu Y Wang C Xiao J. Changed hub and corresponding functional connectivity of subgenual anterior cingulate cortex in major depressive disorder. Front Neuroanat. (2016) 10:120. 10.3389/fnana.2016.00120 28018183 PMC5159433

[45] Smith DV Sip KE Delgado MR. Functional connectivity with distinct neural networks tracks fluctuations in gain/loss framing susceptibility. Hum Brain Mapp. (2015) 36:2743–55. 10.1002/hbm.22804 25858445 PMC4736507

[46] Spechler PA Chaarani B Orr C Mackey S Higgins ST Banaschewski T. Neuroimaging evidence for right orbitofrontal cortex differences in adolescents with emotional and behavioral dysregulation. J Am Acad Child Psy. (2019) 58:1092–103. 10.1016/j.jaac.2019.01.021 31004740

[47] Phillips ML Drevets WC Rauch SL Lane R. Neurobiology of emotion perception I: The neural basis of normal emotion perception. Biol Psychiatry. (2003) 54:504–14. 10.1016/S0006-3223(03)00168-9 12946879

[48] Serafini G. Neuroplasticity and major depression, the role of modern antidepressant drugs. World J Psychiatry. (2012) 2:49. 10.5498/wjp.v2.i3.49 24175168 PMC3782176

[49] Greicius MD Supekar K Menon V Dougherty RF. Resting-state functional connectivity reflects structural connectivity in the default mode network. Cerebral cortex. (2009) 19:72–8. 10.1093/cercor/bhn059 18403396 PMC2605172

[50] Chen Y Wang C Zhu X Tan Y Zhong Y. Aberrant connectivity within the default mode network in first-episode, treatment-naive major depressive disorder. J Affect Disord. (2015) 183:49–56. 10.1016/j.jad.2015.04.052 26001663

[51] Teng S Lu CF Wu YT Wang PS Yeh TC Su TP. Investigation of differences on functional connectivity in major depressive disorder using functional magnetic resonance imaging. In 2010 International Conference on Bioinformatics and Biomedical Technology. IEEE. (2010). p. 115–9.

[52] Rolls ET. The Brain, Emotion, and Depression. Oxford University Press. (2018).

[53] Kong L Chen K Tang Y Wu F Driesen N Womer F. Functional connectivity between the amygdala and prefrontal cortex in medication-naive individuals with major depressive disorder. J Psychiatry Neurosci. (2013) 38:417. 10.1503/jpn.120117 24148846 PMC3819156

[54] Rushworth MF Paus T Sipila PK. Attention systems and the organization of the human parietal cortex. J Neurosci. (2001) 21:5262–71. 10.1523/JNEUROSCI.21-14-05262.2001 11438601 PMC6762864

[55] Ye M Yang T Qing P Lei X Qiu J Liu G. Changes of functional brain networks in major depressive disorder: a graph theoretical analysis of resting-state fMRI. PLoS ONE. (2015) 10:e0133775. 10.1371/journal.pone.0133775 26327292 PMC4556670

[56] Gusnard DA Akbudak E Shulman GL Raichle ME. Medial prefrontal cortex and self-referential mental activity: relation to a default mode of brain function. Proc Nat Acad Sci. (2001) 98:4259–64. 10.1073/pnas.071043098 11259662 PMC31213

[57] Sheline YI Price JL Yan Z Mintun MA. Resting-state functional MRI in depression unmasks increased connectivity between networks via the dorsal nexus. Proc Nat Acad Sci. (2010) 107:11020–5. 10.1073/pnas.1000446107 20534464 PMC2890754

[58] Brakowski J Spinelli S Dörig N Bosch OG Manoliu A Holtforth MG. Resting state brain network function in major depression–depression symptomatology, antidepressant treatment effects, future research. J Psychiatr Res. (2017) 92:147–59. 10.1016/j.jpsychires.2017.04.007 28458140

[59] Wang W Zhao Y Hu X Huang X Kuang W Lui S. Conjoint and dissociated structural and functional abnormalities in first-episode drug-naive patients with major depressive disorder: a multimodal meta-analysis. Sci Rep. (2017) 7:10401. 10.1038/s41598-017-08944-5 28871117 PMC5583354

[60] Jing B Liu C-H Ma X Yan H-G Zhuo Z-Z Zhang Y. Difference in amplitude of low-frequency fluctuation between currently depressed and remitted females with major depressive disorder. Brain Res. (2013) 1540:74–83. 10.1016/j.brainres.2013.09.039 24121137

[61] Zhou M Hu X Lu L Zhang L Chen L Gong Q. Intrinsic cerebral activity at resting state in adults with major depressive disorder: a meta-analysis. Prog Neuropsychopharmacol Biol Psychiatry. (2017) 75:157–64. 10.1016/j.pnpbp.2017.02.001 28174129

[62] Schmahmann JD. Disorders of the cerebellum: ataxia, dysmetria of thought, and the cerebellar cognitive affective syndrome. J Neuropsychiatry Clin Neurosci. (2004) 16:367–78. 10.1176/jnp.16.3.367 15377747

[63] Phillips JR Hewedi DH Eissa AM Moustafa AA. The cerebellum and psychiatric disorders. Frontiers in public health. (2015) 3:66. 10.3389/fpubh.2015.00066 26000269 PMC4419550

[64] Landin-Romero R Novo P Vicens V McKenna PJ Santed A Pomarol-Clotet E. EMDR therapy modulates the default mode network in a subsyndromal, traumatized bipolar patient. Neuropsychobiology. (2013) 67:181–4. 10.1159/000346654 23548794

[65] Pagani M Di Lorenzo G Verardo AR Nicolais G Monaco L Lauretti G. Neurobiological correlates of EMDR monitoring–an EEG study. PLoS ONE. (2012) 7:e45753. 10.1371/journal.pone.0045753 23049852 PMC3458957

[66] Matsuo K Kasai K Kato T Kato N. Hemodynamic responses of eye movement desensitization and reprocessing in posttraumatic stress disorder. Neurosci Res. (2009) 65:375–83. 10.1016/j.neures.2009.08.014 19729044

